# Interplay between Exosomes and Autophagy in Cardiovascular Diseases: Novel Promising Target for Diagnostic and Therapeutic Application

**DOI:** 10.14336/AD.2018.1020

**Published:** 2019-12-01

**Authors:** Jinfan Tian, Mohammad Sharif Popal, Yingke Zhao, Yanfei Liu, Keji Chen, Yue Liu

**Affiliations:** ^1^Department of Cardiology, Beijing Anzhen Hospital, Capital Medical University, Beijing, China; ^2^Cardiovascular disease center, Xiyuan Hospital, China Academy of Chinese Medical Sciences, Beijing, China; ^3^Li Ka Shing Faculty of Medicine, The University of HongKong, Pokfulam, Hong Kong.; ^4^Graduate School, Beijing University of Chinese Medicine, Beijing, China

**Keywords:** exosomes, autophagy, cardiovascular disease, atherosclerosis, myocardial ischemia/reperfusion injury

## Abstract

Exosome, is identified as a nature nanocarrier and intercellular messenger that regulates cell to cell communication. Autophagy is critical in maintenance of protein homeostasis by degradation of damaged proteins and organelles. Autophagy and exosomes take pivotal roles in cellular homeostasis and cardiovascular disease. Currently, the coordinated mechanisms for exosomes and autophagy in the maintenance of cellular fitness are now garnering much attention. In the present review, we discussed the interplay of exosomes and autophagy in the context of physiology and pathology of the heart, which might provide novel insights for diagnostic and therapeutic application of cardiovascular diseases.

## 1. Introduction

Currently, it has been established that the messenger functions of exosomes are critical in neointimal formation, vascular repair, and atherosclerosis [1-3]. Moderate autophagy exerts protective role in cardiovascular diseases (CVDs)[4, 5]. Evidence has disclosed that the existence of a crosstalk between exosomes and autophagy in the protection of cellular homeostasis [6-8]. Exosomes contain molecular signals that regulate autophagy, which in turn, regulate exosomal activities [6, 9, 10].

**Figure 1.**
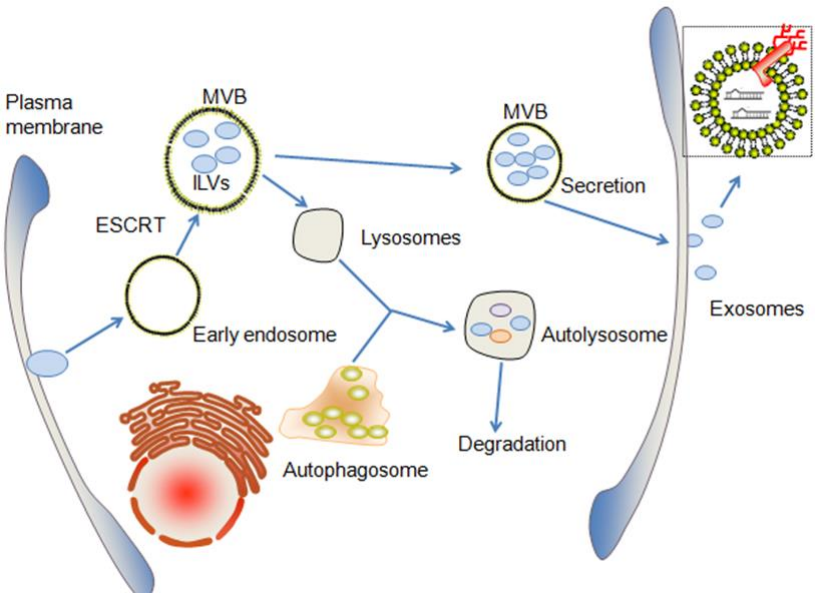
The crosstalk between autophagic and exosomal process. MVBs which enrich in ILVs generates from early endosomes directed by ESCRT. MVBs can either fuse with lysosomes, which subsequently fuse with auto-phagosome to form autolysosome leading to autophagic degradation, or MVBs could fuse with the plasma membrane to release vesicles as exosomes into the extracellular space. Exosomes contains rich biological signaling materials as non-coding RNAs. ESCRT, endosomal sorting complex required for transport; ILVs, intraluminal vesicles.

Extracellular vesicles like micro-vesicles and apoptotic bodies serve as intercellular messengers. The early endosomes were generated from plasma membrane through inward of buds under the stimulation of physical and or chemical factors. The late endosomal membrane inward buds and produces multivesicular bodies named MVBs, directed by the machinery of endosomal sorting complex required for transport (ESCRT), eventually leading to the accumulation of intraluminal vesicles (ILVs) within MVBs [1]. MVBs can either fuse with lysosomes, which subsequently fuse with auto-phagosome to form autolysosome leading to autophagic degradation, or fuse with the plasma membrane to release vesicles, such as exosomes, into the extracellular space [6] (Fig. 1).

The endosomal and autophagic procedures are consequences of the biogenesis of exosomes which depend on the cellular activity of autophagy [6, 7]. The suppression of autophagosome maturation or fusion with a lysosome commonly results in inadequate digestion of damaged proteins or organelles, leading to progressive accumulation of deleterious material, might promote exosomal procedure to release the partially digested or undigested materials [6, 7, 10]. Alternatively, under cellular starvation conditions, the bio-balance would be shifted towards greater autophagic degradation, and a reduced biogenesis of exosomes [6, 7, 10]. Taken together, induction of the autophagy pathway has been shown to decrease exosomal release by promoting fusion of MVBs with autophagosomes, while impaired autophagy may lead to increase exosomes secretion. ILVs enrich in biological materials, including messenger RNA (mRNA), microRNA (miRNA), and small interfering RNA (siRNA). Exosomes transfer cell-cell communication and capable of regulating autophagy by these biological molecules [11, 12]. This review for the first time discussed the interplay of exosomes, autophagy and CVDs.

## 2. Exosomes-related non-coding RNAs and CVDs

The roles of exosomes in CVDs meticulously depend on the type of cell it is derived from [1, 13]. Correspondingly, exosomes-related non-coding RNAs exert various functions in the progression of CVDs [14] (Table 1).

**Table 1:** Overview of exosomes-related non-coding RNAs in cardiovascular disease

non-coding RNA	Cardiovascular disease	Cell types	Effects
IncRNA GAS5	Atherosclerosis↑	THP-1 cells	The apoptosis of vascular endothelia cells↑ [16]
miR-143/145	Atherosclerosis↓	Endothelial cells	Communication between endothelial and smooth cells↑ [17]
miR-155	Atherosclerosis↑	Vascular smooth muscles cells	Endothelial permeability↑ [18]
miR-223, miR-339 and miR-21	Atherosclerosis↓	Platelet	NF-κB pathways↓ [19, 20]
miR-21	Myocardial infarction↓	EnMSCs	Cardiomyocytes apoptosis↓ [36]
miR-126	Myocardial ischemia/reperfusion injury↓	Adipose-derived stem cells	Apoptosis, inflammation, fibrosis↓angiogenesis↑ [37]
miR-93-5p	Myocardial infarction ↓	Adipose-derived stromal cells	Hypoxia-induced autophagy and inflammation↓ [11]
miR-451	Myocardial ischemia/reperfusion injury↓	Cardiac progenitor cells	Oxidative stress-induced apoptosis↓ [40]
miR-21	Myocardial ischemia/reperfusion injury↓	Cardiac progenitor cells	Oxidative stress-induced apoptosis↓ [41]

EnMSCs, MSCs derived from the endometirum

### 2.1 Exosomes-related non-coding RNAs and atherosclerosis

It has been established that atherosclerosis is characterized by ox-LDL induced inflammation, endothelial injury, apoptosis and phonotype changes of vascular cells and platelet activation. Most recent studies demonstrated that, the exosomal pathway for communication of macrophages and vascular endothelial cells modulates the apoptosis and inflammation response, participating in the pathophysiology of atherosclerosis [3, 14, 15, 16]. Furthermore, the communication between endothelial cells and smooth muscle cells mediated by exosomes affects the inflammation and phonotype changes of vascular cells, which are closely associated with the progression of arthrosclerosis [17]. Huang C *et al.* [15] revealed that, inhibiting the secretion of exosomes by ox-LDL-stimulated macrophages restored the growth and tube formation of endothelial cells. The findings of Chen L *et al.* [16] revealed that exosomes derived from IncRNA GAS5 over-expressed THP-1 cells enhanced the apoptosis of vascular endothelia cells, suggesting that exosomes-associated IncRNA GAS5 regulated the apoptosis of macrophages and endothelial cells. According to Eduard Hergenreider E *et al.* [17], extracellular vesicles derived from KLF2-expressing endothelial cells which enrich in miR-143/145 reduced atherosclerotic lesion formation in the aorta of ApoE-/- mice by increased communication between endothelia cells and smooth muscle cells through miRNA- and extracellular-mediated mechanism. Study from Zheng B *et al.* [18] revealed that the transfer of Krüppel-like factor 5 (KLF5)-induced miR-155 from Smooth muscle cells (SMCs) to endothelial cells mediated by vascular smooth muscle cells (VSMCs)-derived exosomes, contributed to impaired tight junctions and the integrity of endothelia barriers, leading to the increased endothelial permeability and enhancing atherosclerotic progress.

Platelet activation and endothelial damage take pivotal roles in atherosclerosis. The findings of Li J *et al.* [19] revealed that the levels of miR-223, miR-339 and miR-21 were elevated in thrombin-activated platelet-derived exosomes. The expression of intercellular adhesion molecule-1 (ICAM-1) stimulated by Tumor necrosis factor-α (TNF-α) in human umbilical vein endothelia cells (HUVECs) was significantly inhibited by miR-223 transfection via inhibition of the phosphorylation of p38 mitogen-activated protein kinase (MAPK), c-Jun N-terminal kinase (JNK) and extracellular regulated protein kinases (ERK), and downregulation of NF-κB pathway. The finding suggested that thrombin-activated platelet-derived exosomes exerted protective effects against atherosclerosis and endothelial inflammation. The downregulating effect of exosomes on ICAM-1 expression was reversed by miR-223 inhibitor. Tan M *et al.* [20] verified that platelet-derived exosomes containing miR-223, miR-339 and miR-21 could be transferred into smooth muscle cells, besides that, it can inhibit platelet-derived growth-factor receptor-beta-stimulated smooth muscle cells proliferation. These findings provide evidence that platelet-derived exosomes could be a promising target for atherosclerosis.

Recently, other types of cells are also reported to be closely related to atherosclerosis. Insulin resistance adipocyte-derived exosomes has been shown to promote plaque burden and plaque vulnerability partly by inducing vasa vasorum angiogenesis in diabetic ApoE-/- mice [21]. Gao W *et al.* [22] observed that mature dendritic cells-exosomes increased HUVECs inflammation via NF-κB pathway in vivo, they further found that the atherosclerotic lesions in ApoE-/- mice was significantly increased by a period of mature dendritic cells injection into ApoE-/- mice. These findings provide novel targets for the management of atherosclerosis.

### 2.2 Exosomes-related non-coding RNAs in acute myocardial infarction

Evidence showed that plasma levels of exosomes-related miRNAs could serve as a novel biomarker for diagnosis of acute myocardial infarction (AMI) [23, 24]. According to Corsten MF *et al.* [25] circulating miRNA-208b were extremely elevated in plasma among AMI patients. Interestingly, miR-499-5p was reported decreased in the infracted area of rat heart, whereas the plasma level was elevated both in AMI rats and patients [26, 27]. Thus, serum miR-449-5p serves as a biomarker for diagnosis of AMI [26-28]. Yang Y *et al.* [29] found that the level of miR-30a increased in the serum of AMI patients in a time-dependent manner. Relative increase of circulating miR-122-5p, as reflected by a high miR-122-5p/133b ratio, is a predictive biomarker to identify patients who at higher risk of an adverse prognosis after ST-elevation myocardial infarction (STEMI). The power of early prognostic value is increased when evaluated in association with left ventricular ejection fraction (LVEF)[30].

Furthermore, preclinical studies also revealed Mesenchymal stem cell (MSC)-derived exosomes exerts beneficial effects for the AMI treatment [31, 32]. MSC-exosomes improved cardiac function by stimulating neovascularization and inhibiting the inflammation response [33]. Arslan F *et al.* [31] showed that bone marrow MSC-derived exosomes reduced infarct size, enhanced myocardial viability and prevented adverse remodeling after ischemic/reperfusion injury in Sprague-Dawley rats via activation of phosphatidylinositol 3-kinase (PI3K)-Akt pathway. According to Zhang Z *et al.* [34], bone marrow MSC-exosomes, containing a large number of bioactive compounds, stimulated the proliferation and angiogenic potency of cardiac stem cells (CSCs). In a rat myocardial infarction model, bone marrow MSC-derived exosomes-preconditioned CSCs increased survival, enhanced capillary density, reduced cardiac fibrosis and restored long-term cardiac function. Wang XL *et al.* [35] revealed that, exosomes derived from human umbilical cord mensenchymal stem cells increased myocardial repair in Sprague-Dawley rats by promoting Smad 7 expression via inhibiting miR-125b-5p. KAN WANG *et al.* [36] for the first time reported that the superiority of MSCs derived from the endometirum (EnMSCs) in anti-MI therapy over those derived from bone marrow or adipose tissue. Exosomal miR-21 that releases from EnMSCs ameliorates cardiac function by regulating cell apoptosis and angiogenesis, and the benefits could be abolished through knockdown of miR-21. These results indicate that MSC-derived exosomes are a potential candidate for adjunctive therapy for patients suffering from acute myocardial infarction.

In addition, the therapeutic value of other type cells derived exosomes for myocardial infarction and the involved mechanisms were investigated in the recent years. According to Luo Q *et al.* [37], exosomes from miR-126-overexpressing adipose-derived stem cells prevented myocardial damage in a rat model via inhibiting apoptosis, inflammation, fibrosis and increased angiogenesis. Adipose-derived stromal cells (ADSC)-derived miR-93-5p-containging exosomes attenuates the infarction-induced rat myocardial damage by inhibition hypoxia-induced autophagy and inflammation through targeting Atg7 and TLR4 respectively [11]. Another study also revealed that miR-145-5p protects H9C2 cardiac cells from hypoxia-induced inflammatory response and apoptosis [38]. V Vicencio JM *et al.* [39] showed that plasma exosomes protect the myocardium from ischemia-reperfusion injury via a HSP70/Toll-like receptor 4 (TLR4) communication pro-survival signaling. Cardiac progenitor (CPC)-derived exosomes emerged as one of the targets for cardiac protection. Chen L *et al.* [40] for the first time showed that cardiac progenitor-derived exosomes (CPC-exosomes), which maintains a high-level expression of GATA4-responsive-miR-451, protected H9C2 myocardial cells from oxidative stress by inhibiting caspase 3/7 activation *in vitro.* Furthermore, CPC-exosomes inhibited cardiomyocyte apoptosis by about 53% compared to PBS control in an acute mouse myocardial ischemia/reperfusion model. Xiao J *et al.* [41] reported that, CPC-exosomes miR-21 played an inhibitory role in the apoptosis pathway through downregulating programmed cell death 4 (PDCD4). Restored miR-21/PDCD4 pathway utilizing CPC-derived exosomes could protect H9C2 cells against oxidative stress-related apoptosis.

### 2.3 Exosomes-related non-coding RNAs and Cardiomyopathy

Myocardial fibrosis and remodeling are pathophysiological mechanisms for cardiomyopathy. De Gonzalo-Calvo D *et al.* [42] first demonstrated that serum levels of cardiomyocyte-enriched miRNA-1 and miR-133a independently predict myocardial steatosis in patients with well-controlled and uncomplicated type 2 diabetes of short duration. Wang X *et al.* [43] disclosed that elevation of Hsp20 in cardiomyocytes offer protection in diabetic mice hearts through the release of instrumental exosomes.

**Table 2:** Exosomes-related miRNAs regulate ABCA1.

miRNA	Effects
miR-33	ABCA1↓ [56-59]
miR-758	ABCA1↓ [61]
miR-20a/b	ABCA1↓ [62]
miR-19b	ABCA1↓ [63]
miRNA-302a	ABCA1↓ [64]

ABCA1, ATP-binding cassette transporter A1

## 3. Exosomes-related miRNA regulates Autophagy in CVDs

Autophagy is an intracellular metabolic self-degradative process regulated by more than 30 highly conserved autophagy-related genes (ATG), in which unwanted cytoplasmic proteins and damaged organelles are degraded by lysosomes. Moderate autophagy is essential for cell homeostasis, while excess of autophagy triggers cell death, indicating that autophagy has both the protective and detrimental functions in the pathological settings [4, 44, 45]. Autophagy is established as a double-edged sword in CVDs [4, 46]. MiRNAs have been exposed to regulate autophagy through post-transcriptional repression of Atg or upstream effectors (BECN1, mTOR, and ULK1) [47, 48]. Vulnerable plaques are characterized by lipid-filled necrotic core resulted from accumulation of apoptotic cells and defective phagocytic clearance [49]. Accumulating evidence supported that macrophages autophagy is an emerging target for atherosclerosis [4, 50-53]. The protective function of autophagy against atherosclerosis associated with cholesterol metabolism and lipophagy. Autophagy facilitates macrophage cholesterol efflux mediated by an ATP-binding cassette transporter A1 (ABCA1)-dependent pathway [54]. The inducing of autophagy via Adenosine 5'-monophosphate (AMP)-activated protein kinase (AMPK) activation is one of the mechanisms by which statins lower plasma cholesterol [55]. Collectively, exosomes-related miRNA regulates autophagy and cholesterol efflux by modulating the expression of ABCA1 (Table 2). MiR-33 represses the expression of ABCA1, reduces cholesterol efflux. On the contrary, inhibition of miR-33 enhances ABCA1 expression, and circulating HDL levels, and improves cholesterol efflux from macrophages, leading to attenuation of atherosclerosis [56-59]. Ouimet M *et al.* [60] provided an insight into the mechanism by which miRNA regulates cellular cholesterol homeostasis and atherosclerosis. The study showed that macrophages treated with anti-miRNA-33 increased efferocytosis, lysosomal biogenesis, and degradation of apoptotic material clearance. Treating atherosclerotic LDL receptor deficiency (LDLr-/-) mice with anti-miR-33 restored defective autophagy in macrophage foam cells and plaques. Ramirez CM *et al.* [61] showed that miR-758 repressed the expression of ABCA1, on the contrary, the inhibition of miR-758 increased ABCA1 expression. According to Liang B *et al.* [62], miR-20a/b inhibited ABCA1 expression, decreased cholesterol efflux and raised cholesterol content in THP-1 and RAW 264.7 macrophage-derived foam cells, and these effects could be reversed by miR-20a/b inhibitors. Lv YC *et al.* [63] presented that miR-19b suppressed ABCA1-dependent cholesterol efflux. Svenja Meiler *et al.* [64] found that long-term in vivo administration of anti-miRNA-302a increased the expression of ABCA1 in the liver but reduced the atherosclerotic lesions in atherogenic diet fed LDLr^-/-^ mice.

## 4. Interplay of Exosomal and Autophagic Pathways, and CVDs

There were two main pathways regulating autophagy, namely, PI3K-Akt-mammalian rapamycin target protein (mTOR) and AMPK-mTOR [65]. PI3K/Akt and mTOR activation are responsible for downregulated autophagy, and AMPK activation inhibits mTOR contributing to upregulated autophagy likewise the function of rapamycin [65]. Currently, the crosstalk between exosomal and autophagic pathways has been reported in a growing number of diseases. The effects of exosomal process on autophagic activity are not completely consistent, maybe partly since the regulation effects of exosomes-related non-coding RNAs on the autophagic signaling pathways varies among the current studies. According to Li L *et al.* [66], overexpression of miR-221/222 inhibited the level of PTEN and activated Akt signaling, and subsequently reduced the expression of hallmarks that positively relate to autophagy including LC3Ⅱ, ATG5 and Beclin1, and increased the expression of SQSTM1/p62. MiR-221/222 from human aortic smooth muscle cells (HAoSMCs)-derived exosomes, inhibited autophagy in HUVECs by modulating PTEN/Akt signaling pathway. The cardio-protective effects of EnMSCs are probably owing to reduced expression of PTEN and increased phosphorylation of Akt by stimulation of exosomal miR-21[36]. In addition, Suzuki T *et al.* [67] also showed that MSC- exosomes decreased autophagy by regulation of mTOR and autophagy-associated protein-13 (ATG13). Liu X *et al.* [68] revealed that miRNA-223 attenuated hypoxia-induced apoptosis and excessive autophagy in neonatal rat cardiomyocytes and H9C2 cells, via Akt/mTOR pathway by targeting poly(ADP-ribose) polymerase 1 (PARP-1). Similarly, Arslan F et al.[31]showed that MCS-exo exerts cardio-protection effect mediated by activation of PI3K/Akt pathway. miR-130a and miR-214 attenuate cardiac dysfunction by activation of PI3K/Akt signaling and downregulation of PTEN expression [69, 70]. However, according to Liang Liu *et al.* [71], treated H9C2 cells with MSCs-derived exosomes enhanced autophagy via AMPK/mTOR and Akt/mTOR pathways. In vivo, exosomes reduced apoptosis and the myocardial infarct size in rats underwent ischemic/reperfusion injury by upregulating LC3B expression. Yang Y *et al.* [29] reported that, inhibition of exosomal miR-30a could attenuate apoptosis of cardiomyocytes induced by hypoxia via augmentation of autophagy, characterized by enhanced expression of core autophagy regulators beclin-1, Atg12, and LC3Ⅱ/Ⅰ, suggesting a novel pathway of autophagy in cardiomyocytes after hypoxia.

## 5. Conclusions and Prospective

Autophagy and exosomes maintain cell homeostasis and fitness in a coordinated manner by removal or secretion harmful substance. The crosstalk between autophagic and exosomal pathways, and the property of exosomes and drug nanocarriers make the donation of exosomes as a cardio-protective approach along with the therapeutic potential for cardiac regeneration [72, 73, 74]. For the evidences that autophagy is an emerging target for CVDs, and tradition Chinese herbal medicines have been demonstrated to be beneficial in treating CVDs through modulation of autophagy [46, 50, 52, 75, 76]. Hence, it is supposed that the MSCs-derived exosomes loaded with traditional Chinese medicine supplement could manipulate medicine-targeting autophagy, a promising therapeutic approach for CVDs [77]. On the other hand, the therapeutic potential of MSCs-derived eoxomes makes the possibility to use exosomes in-situ tissue regeneration and remodeling [78-81].
